# Physics-Aware Ensemble Learning for Superior Crop Recommendation in Smart Agriculture

**DOI:** 10.3390/s25196243

**Published:** 2025-10-09

**Authors:** Hemalatha Gunasekaran, Krishnamoorthi Ramalakshmi, Saswati Debnath, Deepa Kanmani Swaminathan

**Affiliations:** 1 College of Computing and Information Sciences, University of Technology and Applied Sciences, Ibri 516, Oman; 2 Centre of Excellence in Computer Vision, Alliance University, Bengaluru 562106, India; debnath.saswati123@gmail.com; 3 Department of Information Technology, Sri Krishna College of Engineering and Technology, Coimbatore 641008, India; deepakanmanis@skcet.ac.in

**Keywords:** smart agriculture, IoT sensors, machine-learning models, ensemble models, physics informed neural network model

## Abstract

Agriculture remains the backbone of many countries; it plays a pivotal role in shaping a country’s overall economy. Accurate prediction in agriculture practices, particularly crop recommendations, can greatly enhance productivity and resource management. IoT and AI technologies have great potential for enhancing precision farming; traditional machine learning (ML) and ensemble learning (EL) models rely primarily on the training data for predictions. When the training data is noisy or limited, these models can result in inaccurate or unrealistic predictions. These limitations are addressed by incorporating physical laws into the ML framework, thereby ensuring that the predictions remain physically plausible. In this study, we conducted a detailed analysis of ML and EL models, both with and without optimization, and compared their performance against a physics-informed ML model. In the proposed stacking physics-informed ML model, the optimal temperature and the pH for each crop (physics law) are provided as input during the training process in addition to the training data. The physics-informed model was trained to simultaneously satisfy two objectives: (1) fitting the data, and (2) adhering to the physics law. This was achieved by including a penalty term within its total loss function, forcing the model to make predictions that are both accurate and physically feasible. Our findings indicate that the proposed novel stacking physics-informed model achieved a highest accuracy of 99.50% when compared to ML and EL models with optimization.

## 1. Introduction

Agriculture is vital to any country, which significantly contributes to its GDP and strengthens the economy. A nation thrives when it can successfully meet its agricultural needs, become self-sufficient, and reduce reliance on other countries for daily necessities [1]. The wealth of a country lies in its farming industry and its farmers. However, modern agriculture faces numerous challenges, including global warming, wars, infectious diseases, and pests. To combat these issues, AI can be used in crop prediction, weather forecasting, soil health analysis, precision farming, yield prediction, pest control, and many more [2,3].

Smart farming leverages IoT sensors to collect real-time and granular data on critical soil and environmental parameters, such as soil nutrients including Nitrogen (N), Phosphorus (P), Potassium (K), soil pH, moisture, temperature, and rainfall [4,5]. These precise measurements enable a deeper understanding of soil health and growing conditions, allowing for data-driven decision-making in agriculture. Using this sensor data, artificial intelligence (AI) and machine learning (ML) techniques are employed to develop crop recommendation systems that advise farmers on the most suitable crops for their fields, thereby optimizing productivity and yield.

Traditional ML models like Decision Trees, K-Nearest Neighbor (KNN), Support Vector Machine (SVM), Gradient Descent, and Naïve Bayes Classifier, often enhanced with sophisticated feature selection and hyperparameter optimization techniques, are employed in developing crop prediction models [6,7,8,9]. Ensemble methods like Random Forest (RF), XGBoost, Gradient Boosting Trees (GBT), and Extra Trees classifiers, which are highly valued for their predictive accuracy and robustness, are also used for crop predictions [6]. Deep learning models such as Convolutional Neural Networks (CNNs) and Long Short-Term Memory (LSTM) networks are widely used for crop predictions [10,11,12]. These models predominantly rely on common environmental and soil-related features such as temperature, rainfall, and soil type, often supplemented by meteorological conditions, nutrient content, and real-time IoT sensor data.

To maximize the performance of these models, hyperparameter optimization methods such as GridSearchCV, RandomSearchCV, and, more recently, automated optimization frameworks like Optuna, have been employed [13,14]. These techniques systematically search the parameter space to identify the combination that delivers the highest predictive accuracy for crop recommendation.

Despite these advances, most existing models are purely data-driven and rely heavily on environmental and soil-related features such as temperature, rainfall, soil type, and nutrient content. While these models achieve high predictive accuracy, they often lack domain-specific interpretability and may overfit to specific datasets, limiting their robustness in diverse real-world environments. Moreover, very few approaches explicitly incorporate physics- or domain-aware constraints (e.g., agronomic knowledge of crop-specific optimal ranges for soil nutrients, pH, and rainfall) into feature engineering.

To address these limitations, this study proposes a physics-aware crop recommendation system that integrates domain knowledge of crop-specific optimal ranges into the feature engineering process. We hypothesize that embedding these domain-specific constraints into the model will not only improve classification accuracy but also enhance generalization and robustness, thereby providing reliable and interpretable recommendations for practical agricultural use [15].

## 2. Literature Review

Accurate crop recommendations based on soil nutrients are crucial for efficient agriculture and maximum yield. Traditional methods rely on soil nutrient analysis and other factors to guide crop recommendations. However, integrating IOT with machine learning has opened up new opportunities to address the limitations of existing methods in crop recommendation. This modern approach allows for more precise, data-driven insights, thereby improving decision-making and agricultural outcomes.

In recent years, there has been significant growth in predicting the type of crop for maximum yield through the application of ML and DL models. Dey et al. (2024) [8] divided the dataset into two parts: one containing 11 agricultural plants and the other containing 10 horticultural plants. The authors implemented five distinct machine learning models—SVM, XGBoost, RF, KNN, and DT—on each of the separate datasets, rather than on a combined dataset. The XGBoost model achieved an accuracy of 99.09%. Islam et al. (2023) [9] employed ML-based algorithms like catBoost, voting, and Bagging to predict the recommended crop. CatBoost obtained the highest accuracy of 97.5%. Kiruthika et al. (2023) [11] proposed a method based on Improved Distribution-based Chicken Swarm Optimization (IDCSO) with weight-based Long Short-Term Memory (WLSTM) for crop prediction. The author achieved an accuracy of 95% by employing IDCSO algorithm for feature selection. Ramzan et al. (2024) [6] implemented ML and EL models on two types of data: real-time data and hybrid data. The author implemented ML algorithms to predict the recommended crop and compare the performance of ML and EL models. Bakthavatchalam et al. (2022) [16] aimed to improve precision agriculture using supervised learning algorithms implemented in WEKA and achieved an accuracy of 98.2273%.

Elbasi et al. (2023) [17] used fifteen different ML algorithms with a new feature combination scheme. The authors achieved 99.59% accuracy using the Bayes Net Algorithm and 99.46% using the Naïve Bayes Classifier and Hoeffding Tree algorithm. S.P. Raja et al. (2022) [18] developed a range of feature selection and classification techniques to predict the yield size of plant cultivations. Sharma et al. (2024) [19] demonstrated how different ML models, such as K-Nearest Neighbors (KNN) and deep learning algorithms, can achieve high accuracy in crop selection and disease prediction. Parween et al. (2021) [20] explored the integration of IoT with ML techniques to create a precise crop prediction system, improving decision-making for farmers through real-time environmental monitoring.

Despite the progress in ML and DL models, precision farming faced some pitfalls due to the scarcity of data availability and as models used for predictions are purely data-driven. The predictions of these models are purely based on the pat2terns found in the training data. If the data is noisy or does not cover all the scenarios, the model may make unrealistic predictions. The physics-informed model addresses this limitation by integrating known physical laws during the training process such as including a crop’s ideal temperature and pH range into the training process.

## 3. Methodology

In this research paper, we have developed a classification model to predict the recommended crop based on seven features: N, P, K, soil pH, temperature, humidity, and rainfall. We have implemented ML models, including Linear Regression (LR), Support Vector Machine (SVM), Decision Tree (DT), Naive Bayes (NB), and K-Nearest Neighbor (KNN). Additionally, we have utilized ensemble learning (EL) models such as Random Forest (RF), Extra Trees, XGBoost, and stacking. These models are optimized using GridSearchCV, RandomSearchCV, and Optuna Optimization techniques. Further, we improved predictive accuracy by incorporating knowledge of ideal crop growth conditions into the NN model, which is called the Physics-Informed Neural Network (PINN) model.

### 3.1. Dataset

**Figure 1:**
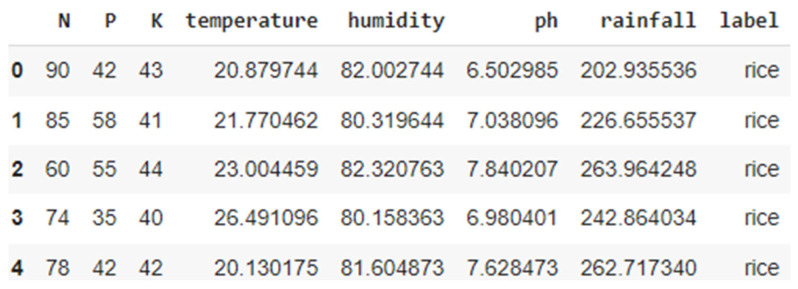
Sample dataset.

**Figure 2:**
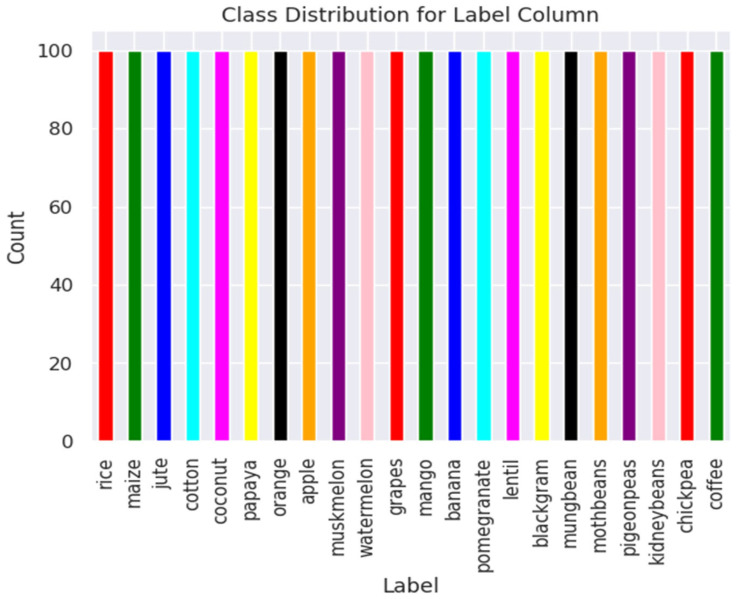
Class distribution of original database.

The dataset used in this study is an IOT sensor dataset available in Kaggle https://www.kaggle.com/datasets/atharvaingle/crop-recommendation-dataset version 1 (accessed on 7 June 2025) [21]. The dataset includes soil nutrient measures like Nitrogen (N), Phosphorus (P), Potassium (K), and other parameters such as pH of soil, moisture, and rainfall, with the type of recommended crop as the target variable, as shown in Figure 1. The Kaggle dataset comprises 2200 records evenly distributed across 22 crop classes, as shown in Table 1, ensuring a balanced representation for each class. The class distribution of the dataset is shown in Figure 2.

### 3.2. Data Preprocessing

The dataset contains feature values measured in a variety of units, reflecting the distinct metrics used for each attribute. N, P and K are measured in kg/hector, temperature measured in °C, humidity measured in %, and rainfall measured in mm. To convert all the features to a common scale, we apply a MinMax scaler to the above-mentioned features.

### 3.3. Proposed Approach

**Figure 3:**
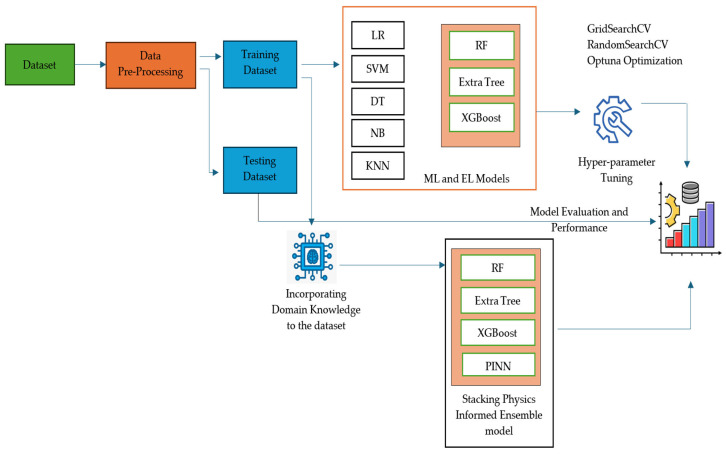
Model of proposed approach.

**Figure 4:**
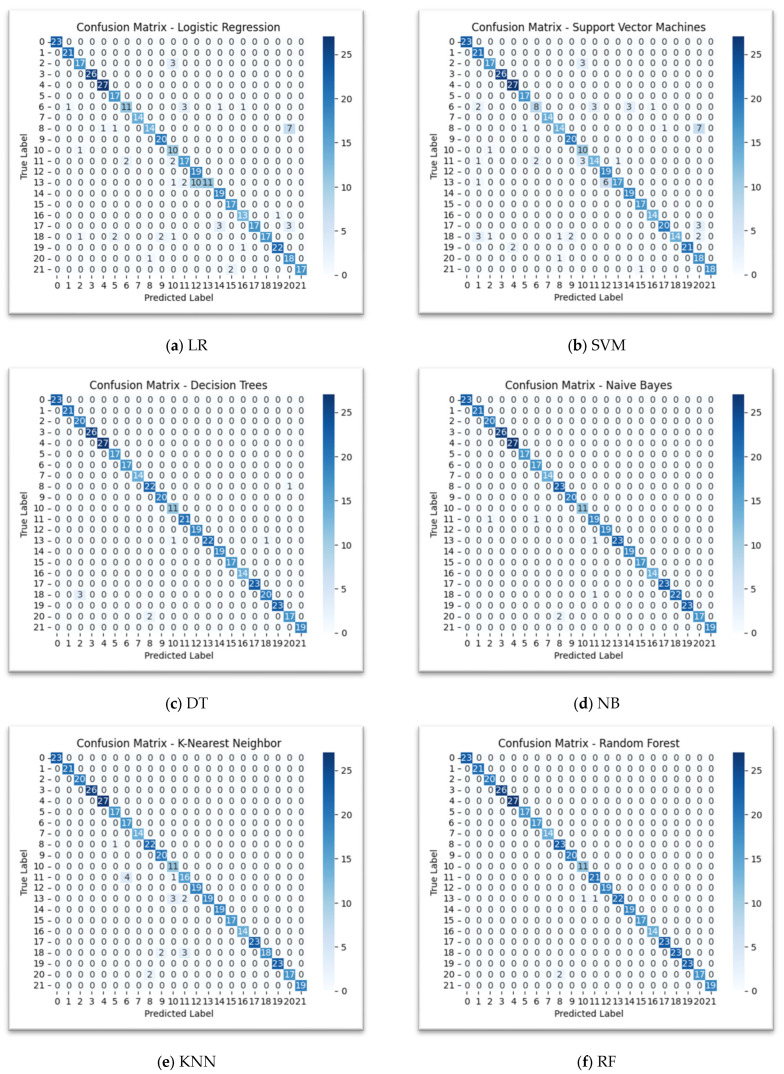

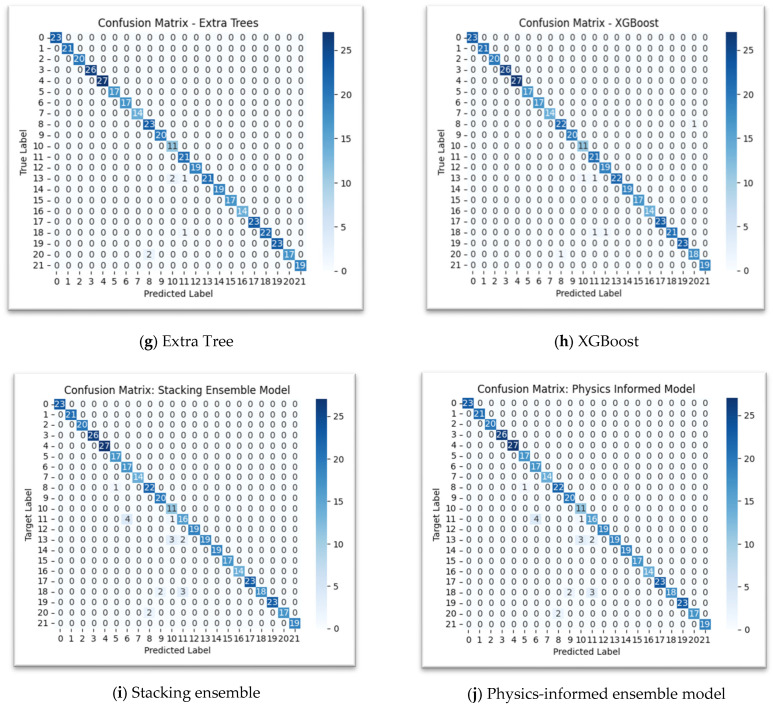
Confusion matrix with Optuna Optimzation.

The preprocessed data is then divided into training and testing datasets of 80% and 20%, respectively, shown in Figure 3. Machine learning models such as LR, SVM, DT, NB, KNN, and stacking ensemble model with RF, Extra Tree, and XGBoost as base estimators and LR as a meta estimator are used to train the model. To ensure our model’s robustness, we then used 5-fold stratified cross-validation. This process, along with the use of a fixed random seed (42), ensures reproducibility. The final model was then tested on the previously held-out 20% test set to provide an unbiased performance estimate. To further improve the model’s performance, we use three optimization techniques, such as GridSearchCV, RandomSearchCV, and Optuna Optimization techniques. The above-mentioned ML and EL models mainly depend on the training dataset; in other words, they are data-driven. The model’s prediction will be unrealistic if the training data is noisy or when the dataset is limited. To overcome these limitations, physics laws are incorporated into the model during the training process (e.g., the ideal values of soil nutrients or environmental factors for each crop are given as input to the model), as shown in Figure 4.

In this research, a stacking physics-informed model is built with RF, Extra Tree, XGBoost, and Physics-Informed Neural Network (PINN) models as base estimators, and LR as meta estimators. The domain knowledge of each crop is incorporated as physics law during the model training process.

### 3.4. Machine Learning Models

#### 3.4.1. Logistic Regression

Logistic Regression is a statistical model used for binary classification tasks. The most crucial hyperparameters for Logistic Regression are ‘C’ and ‘penalty’. ‘C’ signifies the inverse of regularization strength; the smaller C value prevents overfitting, and the larger C value closely fits with the training data, thereby increasing the risk of overfitting, especially with noisy data. The ‘penalty’ hyperparameter takes two values, either ‘l1’ or ‘l2’. ‘l1’ (Lasso) encourages sparsity and it is useful for feature selection (irrelevant features are automatically dropped); it can improve accuracy if the data is noisy data. While ‘l2’ (Ridge) penalizes the squared magnitude of coefficients, shrinking them towards zero without necessarily eliminating them, it helps prevent overfitting by reducing weight.

#### 3.4.2. Support Vector Machine

It is one of the effective approaches for classification and regression tasks and is able to handle high-dimensional data and maintain robustness. SVM seeks to find an optimal hyperplane that best separates data points belonging to different classes in a high-dimensional feature space. Two hyperparameters are selected to tune the model; ‘C’ controls the trade-off between margin width and classification errors. A smaller C value leads to a wider margin, resulting in underfitting (high bias, low variance). A larger C value leads to a narrow margin, resulting in overfitting (low bias, high variance). The parameter ‘max_iter’ specifies the maximum number of iterations the optimization algorithm is allowed to run while fitting the SVM model. Too low a value will stop the model before convergence. Too high a value ensures the algorithm has time to converge.

#### 3.4.3. Naïve Bayes

Tuning a Naïve Bayes model is relatively simple because Naïve Bayes has fewer hyperparameters compared to other models like SVM or DT. The ‘var_smoothing’ parameter helps stabilize training and can change decision boundaries. A smaller value for ‘var_smoothing’ results in overfitting, a larger value results in underfitting. The best value should be close to default $1\;\times \;10^{-9}$ but it should be tuned with cross validation.

#### 3.4.4. K-Nearest Neighbor

KNN is a non-parametric classifier. It predicts the label of a query instance by identifying the k-closest training samples in the feature space and assigning the most frequent class label among them. The closeness is typically measured using a distance metric, specifically Euclidean distance, although other metrics like Manhattan or Minkowski can also be used. The number of neighbors ‘n_neighbors’ is a critical hyperparameter that controls the size of the neighborhood used for majority voting. Smaller ‘n_neighbors’ can overfit, larger ‘n_neighbour’ may unfit. The ‘weight’ hyperparameter decides how neighbors are weighted in voting.

#### 3.4.5. Random Forest

Random Forest is an ensemble learning algorithm that builds multiple Decision Trees and merges their results to achieve a more accurate and stable prediction. It is used for both classification and regression tasks. For classification tasks, the final prediction is determined by a majority vote across all trees in the forest.

The ‘n_estimators’ parameter indicates the number of trees in the forest, by default it is 100; more trees result in better performance but result in high computation cost. The ‘max_depth’ parameter indicates the maximum depth of each tree; by default, it is none. A smaller value results in underfitting, a larger value results in overfitting. The ‘min_sample_split’ parameter indicates minimum samples needed to split a node; by default, it is 2.

#### 3.4.6. Extra Trees Classifier

The Extra Trees Classifier is an ensemble learning method based on the principle of randomized DT. It is very similar to RF but adds more randomness. In RF, the best split is selected among a random subset of features. In Extra Trees, both the features and split thresholds are selected randomly. The key hyperparameters of Extra Trees are the same as RF.

#### 3.4.7. XGBoost

It builds an ensemble of DT in a sequential manner, where each tree tries to correct the errors of the previous one. The key hyperparameters tunned are ‘n_estimators’, which specifies the number of boosting rounds or trees in the ensemble; ‘learning_rate’, which controls the contribution of each new tree to the overall prediction; ‘max_depth’, which limits the maximum depth of each DT; and ‘subsample’, which defines the fraction of the training data to be randomly sampled for each boosting round.

### 3.5. Optimization Techniques

#### 3.5.1. Grid SearchCV

Grid SearchCV is a systematic approach to hyperparameter optimization that evaluates the performance of a model for every possible combination of specified hyperparameter values using cross-validation. This exhaustive search ensures that the best possible combination of parameters is found within the provided grid, resulting in potentially higher model accuracy. However, it can become computationally expensive as the number of parameters and their possible values increase.

#### 3.5.2. Random SearchCV

Random SearchCV is a hyperparameter optimization technique that samples a fixed number of random combinations from a predefined parameter grid and evaluates model performance for each combination using cross-validation [14]. Unlike Grid SearchCV, which exhaustively checks all possible parameter values, Random Search focuses only on a subset, making it more efficient when dealing with a large number of hyperparameters or expansive search spaces.

#### 3.5.3. Optuna Optimization

Optuna is an advanced hyperparameter optimization library that employs state-of-the-art techniques such as Bayesian optimization for efficient and automated parameter search [22,23,24]. Instead of sampling parameters randomly or exhaustively, Optuna uses intelligent trial selection strategies to focus on promising regions of the search space, adaptively learning from past results to improve future suggestions. This approach accelerates the discovery of optimal or near-optimal settings, making it ideal for complex machine learning tasks like crop recommendation. With its flexibility, easy integration, and ability to prune unpromising trials early, Optuna helps achieve highly accurate models while significantly reducing computational overhead.

## 4. Proposed Physics-Informed NN Model

In this research, we propose a stacking ensemble model with four base learners: PINN, RF, Extra Tree, and XGBoost classifiers. The predictions of these base learners are consolidated by a meta-learner (Logistic Regression) to obtain the final prediction.

In PINN model, a standard NN model is built to incorporate a physics-informed loss function in addition to the standard cross-entropy loss [25,26,27,28,29]. It defines three linear layers with ReLU activation functions in between, which help the network learn complex, non-linear patterns. The final layer has an output size equal to the number of crop classes. It contains a dictionary that stores the mean (μ) and standard deviation (σ) of ideal temperature and pH for each crop. This information acts as the physical law. In each epoch, it calculates two types of loss: data loss and physics-informed loss.

The physics-informed classifier calculates a total loss that is a weighted sum of two components: the data loss and the physics-informed loss. It is given by the following formula:

(1) $$L_{Total}\;=\;L_{Data}\;+\;\lambda_{physics}\;\times \;L_{physics}$$

where $\lambda_{physics}$ (lambda_physics) is the hyperparameter that controls the importance of the physics-informed loss relative to data loss. The data loss $L_{Data}$ is the cross-entropy loss that predicts how well the model’s prediction aligns with the true label. This loss function penalizes the model when it predicts a low probability for the correct crop. The cross-entropy loss is calculated as follows:

(2) $$L_{data}=-\frac{1}{N}\sum_{i=1}^{N}\sum_{c=1}^{C}y_{i,c}log(\hat{y_{i,c}})$$

where C is the number of crop classes, $y_{i,c}$ is a binary indicator (1 if true class, 0 otherwise), $\hat{y_{i,c}}$ is the predicted probability of sample *i* belonging to class C.

The physics-informed loss $L_{physics}$ penalizes the model when it predicts a low probability for a crop under its ideal growing temperature and pH. The model is trained with domain knowledge, which contains the mean (μ) and standard deviation (σ) of the ideal temperature and pH for each crop. For each training sample and for each crop, the model calculates a temperature score and pH score using a Gaussian function, as given in Formulas (3) and (4). This score is highest when the training sample’s temperature and pH is very close to the ideal values, and decreases as it deviates from the ideal value.

(3) $$temp\_score=exp(-\frac{{\left(input\_temp-\mu_{Tcrop}\right)}^{2}}{2\sigma_{Tcrop}^{2}})$$

(4) $$pH\_score=exp(-\frac{{\left(input\_pH-\mu_{pHcrop}\right)}^{2}}{2\sigma_{pHcrop}^{2}})$$

where $\mu_{Tcrop}$ and $\sigma_{Tcrop}$ are the mean and the standard deviation of the ideal temperature and $\mu_{pHcrop}$ and $\sigma_{pHcrop}$ are the mean and standard deviation of ideal pH. The physics-informed loss for a single crop is calculated as the product of the model’s confidence and the temperature and pH score, as given in (5).

(5) $$L_{physics}=\left(1-crop_{prob}\right)\times temp_{score}\times {pH}_{score}$$

## 5. Model Evaluation

The experiment was conducted in Google Colab Pro with Python 3 Google Compute Engine backend with 15 GB GPU RAM and 12 GB of system RAM. The dataset is divided into training and testing in a ratio of 80% and 20%, respectively. Seven ML models, such as LR, SVM, DT, NB, KNN, RF, Extra Tree, and XGBoost, are tested on the Kaggle dataset. The model performance is improved by implementing a stacking ensemble model with RF, Extra Tree, and XGBoost as base estimators and LR as the meta estimator. The performance of the model can be improved by applying hyperparameter tuning techniques. Hyperparameters are parameters that control the learning process of a model. Different machine learning models have different hyperparameters. Properly tuned hyperparameters can result in significant improvements in terms of accuracy, efficiency, and generalization of a model to new, unseen data. Conversely, poor selection can lead to overfitting, underfitting, and inefficient training processes. As a result, techniques such as GridSearchCV, RandomSearchCV, and more advanced frameworks such as Optuna are used to find the most effective hyperparameter settings for specific models and datasets. The hyperparameters tuned for each model are given in Table 2.

The models are tested using stratified K-fold cross-validation using a split size of 5 to ensure the models’ evaluation is fair and reliable. The performance of the models is evaluated based on the metrics such as accuracy (6), precision (7) and recall (8), as shown below:

(6) $$Accuracy\;=\;\frac{TP\;+\;TN}{TP\;+\;TN\;+\;FP\;+\;FN}$$

(7) $$Precision\;=\;\frac{TP}{TP+FP}$$

(8) $$Recall\;=\;\frac{TP}{TP+FN}$$

All performance metrics are reported as the mean ± standard deviation. Mean is the best estimate of the model’s overall performance. It is the average scores from all 5 test folds. Standard deviation is the variability of the model’s performance; a low standard deviation means the model performs consistently well across different subsets of the data. A high standard deviation indicates that the model’s performance highly depended on the training data; as a result, it may be less stable and reliable in a real-world scenario.

The results of our comparative analysis demonstrate that hyperparameter optimization is a critical step in maximizing the performance of machine learning classifiers. The best hyperparameter values for all the models are given in Table 3. Across all models tested, a notable improvement in accuracy was observed after the application of optimization techniques. The baseline performance, as shown in Table 4’s ‘Without Optimization’ column, was consistently surpassed by the optimized versions. For instance, the LR model showed the most significant improvement with optimization. Its accuracy increased from 0.8812 ± 0.0182 to 0.9784 ± 0.0109 with Optuna, a gain of over 9%. Similarly, precision and recall scores also showed a major improvement. SVM’s performance also improved with optimization. Its accuracy peaked at 0.9335 ± 0.0167 with Optuna. The Decision Tree model performed well without optimization, with an accuracy of 0.9784 ± 0.0058. However, Grid Search provided a small boost, pushing accuracy to 0.9864 ± 0.0092. The NB model showed remarkably high performance, with an accuracy of 0.9938 ± 0.0028 without optimization. Unlike other models, NB optimization had a minimal effect, suggesting its simple nature already captures the underlying patterns effectively. KNN’s accuracy improved from 0.9733 ± 0.0106 to 0.9744 ± 0.0076 with Grid Search and Random Search, but dropped with Optuna, reaching 0.9727 ± 0.0093. RF model had a strong baseline performance of 0.9938 ± 0.0033 accuracy. Optimization with Grid and Random Search slightly improved the accuracy, but Optuna resulted in a minor drop in performance, similar to KNN. Extra Tree model performed well, with an accuracy of 0.9909 ± 0.0061; with Grid and Random Search, accuracy increased to 0.9915 ± 0.0074 and 0.9920 ± 0.0049, respectively. However, Optuna resulted in a slight decrease in accuracy to 0.9882 ± 0.0046. XGBoost model’s accuracy improved from 0.9847 ± 0.0085 to 0.9914 ± 0.0044 with Optuna Optimization. The stacking ensemble model consistently achieved the highest scores. Without optimization, its accuracy was 0.9923 ± 0.0043; with Optuna, its accuracy boosted to 0.9948 ± 0.0023. This result underscores the power of ensemble methods in leveraging the collective strengths of individual base classifiers.

A comparative analysis of the optimization methods revealed that Optuna Optimization consistently delivered the highest accuracy values across the majority of the models. This suggests that its advanced Bayesian search strategy is more effective and efficient at navigating the complex hyperparameter space than the more exhaustive GridSearchCV or RandomizedSearchCV.

**Figure 5:**
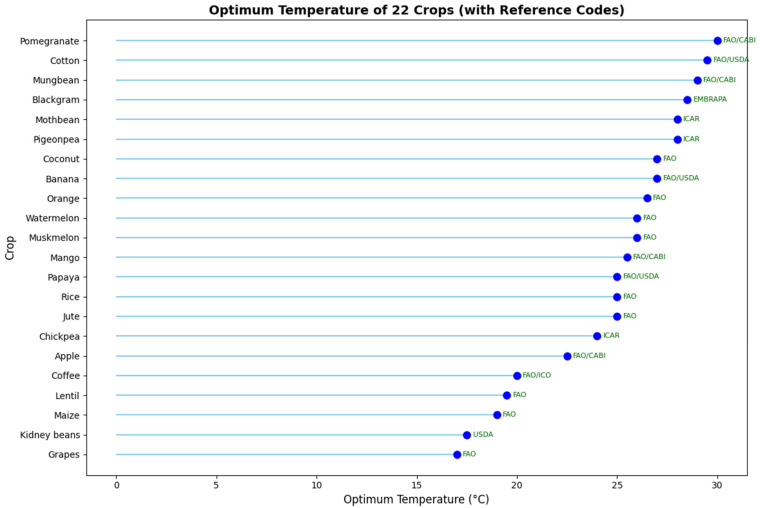
Optimum temperature for all the 22 crops from the agronomic references.

The confusion matrices visually represent the performance of each model by showing the number of correct and incorrect predictions for each class. The diagonal elements, where the “Predicted Label” matches the “True Label,” represent the correct predictions and the off-diagonal elements represent misclassifications. DT, NB, KNN, RF, Extra Trees, XGBoost, stacking, and physics-informed models show a very strong performance, with a high concentration of correct predictions along the main diagonal, as shown in Figure 5. LR shows more misclassifications than the other models. For example, classes 11, 12, 13, and 19 show a few misclassifications, as shown in Figure 4a. The SVM matrix shows a similar pattern to LR, with some misclassifications in classes like 11, 12, 13, and 19.

**Figure 6:**
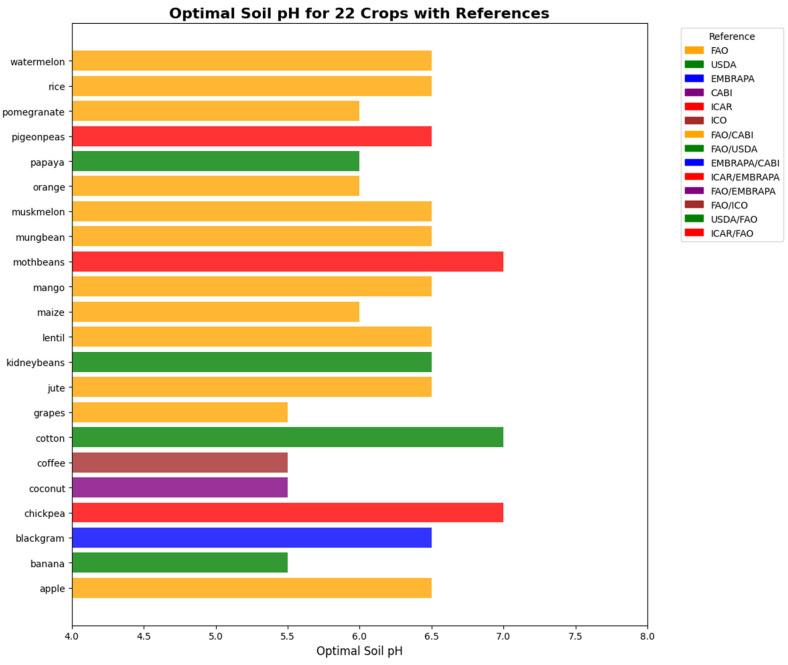
Optimum pH for all the 22 crops from the agronomic references.

In the physics-informed neural network (PINN), the optimal temperature and optimal soil pH for all 22 crops are incorporated as physics-based constraints during training. The optimum value for temperature and pH are shown in Figure 5 and Figure 6 respectively. Due to limitations in obtaining validated optimal values for all agronomic parameters across these crops, only these two parameters were included as physical laws. During training, the model computes both the standard data loss and an additional physics-informed loss. Predictions that deviate from these physical constraints are penalized, and the magnitude of this penalty is controlled by the hyperparameter $\lambda_{physics}$. This approach guides the model to make predictions that are consistent with known agronomic principles, while still learning from the data.

**Figure 7:**
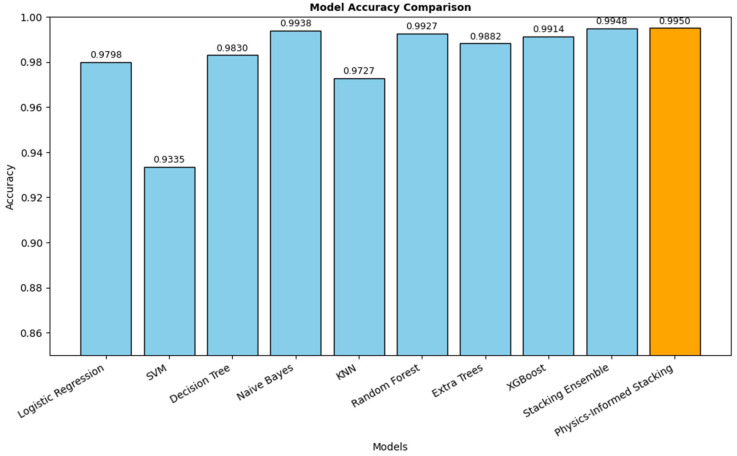
Comparison of classification accuracy for different machine learning models under Optuna Optimization with the proposed model.

The physics-informed stacking ensemble model demonstrated the best overall performance among all tested approaches, achieving an accuracy of 0.9950 ± 0.0017, precision of 0.9952 ± 0.0016, and recall of 0.9950 ± 0.0017 across five-fold cross-validation. Compared with conventional machine learning models, such as LR, SVM, DT, KNN, and XGBoost, the physics-informed ensemble model provides a clear improvement in predictive capability, as shown in Figure 7. Even against the ensemble model, the proposed model achieves slightly higher accuracy while maintaining lower variance, indicating enhanced stability. These findings suggest that the integration of domain-specific physical constraints within the ensemble learning framework not only improves classification accuracy but also yields more robust generalization.

The proposed stacking ensemble model with PINN model is compared with the existing models in Table 5. The proposed model obtained the highest accuracy of 99.50% when compared to all the other existing models in [3,5,6,9,12,13]. The proposed model outperformed several other significant models included in the comparison. It surpassed the XGBoost model (99.09%), the KNN model (97.81%), and the MLP model (98%). The proposed model’s accuracy also significantly exceeds that of the Bagging and IDCSO-WLSTM models, which achieved 97.29% and 92.68% accuracy, respectively. The results indicate that the stacking physics-informed NN model is one of the most accurate solutions for this specific dataset among the models reviewed.

The findings of this study demonstrate the potential of machine learning and physics-informed models to support real-world monitoring and decision-making. Such approaches could be applied in industrial, agricultural, or environmental settings where continuous monitoring is required. Although this study used a limited dataset, the results highlight the feasibility of combining data-driven and physics-informed approaches, with future validation on larger and more diverse datasets needed before large-scale deployment.

## 6. Conclusions

In this research, we compared the performance of standard ML, EL, and PINN models. Our evaluation on the Kaggle crop dataset showed that the EL model, particularly a stacked EL model comprising Extra Tree, RF, and XGBoost Classifier as base learners, with LR as the meta-learner, outperformed other individual ML models, achieving an accuracy of 99.23%. Optimization of the stacked EL model using Optuna further improved the accuracy to 99.48%. By incorporating PINN as one of the base learners in a stacking EL model, the model’s predictive capability can be improved. PINN is built by incorporating the domain knowledge of crop conditions (e.g., optimal temperature) into the model during the training process. The PINN model reached an accuracy of 99.50%, demonstrating the value of integrating scientific knowledge with advanced machine learning methodologies. The results are promising within a benchmark dataset but require validation on real-world data.

## 7. Limitations and Future Work

The database used in this study is relatively limited in size, which may affect the generalizability of the model. The dataset contains no missing values, which is not fully representative of real-world agricultural datasets. In the PINN model, only two optimal features, temperature and soil pH, were included as physics-based constraints.

Incorporating additional optimal agronomic parameters as physics-based constraints can enhance the PINN model’s predictive capability. Introducing noise in the dataset will help to evaluate the robustness and real-world applicability of the model.

## Figures and Tables

**Table 1:** Dataset details.

Kaggle Dataset	
No. of rows	2200
No. of samples per class	100
No. of classes for target variable	22

**Table 2:** Hyperparameters of the model.

Model	Hyperparameters
Logistic Regression	C > 0, penalty [l1, l2]
Support Vector Machines	C > 0, kernel, degree
Decision Trees	max_depth [any positive integer], min_samples_split [any positive integer]
Naive Bayes	priors, var_smoothing
K-Nearest Neighbor	n_neighbors [≥1], weights [uniform’, ‘distance’]
Random Forest	n_estimators, max_features, random_state
Extra Tree Classifier	n_estimators, max_features, random_state
XGBoost	n_estimators, learning_rate, max_depth, subsample

**Table 3:** Hyperparameter values.

Model	GridSearchCV	RandomSearchCV	Optuna
LR	C: 1000, penalty: l2	C: 100, penalty: l2	C: 829.97, penalty: l2
SVM	max_iter: 1000 C: 10	max_iter: 1000 C: 54.555	max_iter: 2000 C: 73.52
DT	min_samples_splits: 2, max_depth: 20	min_samples_splits: 2, max_depth: 30	min_samples_splits: 3, max_depth: 30
NB	var_smoothing: 1 $\times \;10^{-9}$	var_smoothing: 1 $\times \;10^{-9}$	var_smoothing: 3.1334
KNN	n_neighbors: 3, weight: distance	n_neighbors: 3, weight: uniform	n_neighbors: 3, weight: distance
RF	max_depth: none, min_samples_split: 2, n_estimators: 200	max_depth: none, min_samples_split: 2, n_estimators: 268	max_depth: 16, min_samples_split: 10, n_estimators: 116
Extra Tree	max_depth: 10, min_samples_split: 5, n_estimators: 100	max_depth: 30, min_samples_split: 2, n_estimators: 268	max_depth: 16, min_samples_split: 5, n_estimators: 441
XGBoost	learning_rate: 0.1, max_depth: 3, n_estimators: 200	learning_rate: 0.3049, max_depth: 3, n_estimators: 180	learning_rate: 0.0640, max_depth: 7, n_estimators: 465

**Table 4:** Performance metrics.

Model	Without Optimization			With GridSearchCV			With RandomSearchCV			With Optuna		
	Accuracy	Precision	Recall	Accuracy	Precision	Recall	Accuracy	Precision	Recall	Accuracy	Precision	Recall
LR	0.8812 ± 0.0182	0.9021 ± 0.0179	0.8800 ± 0.0183	0.9710 ± 0.01454	0.9732 ± 0.0130	0.9710 ± 0.0145	0.9767 ± 0.0104	0.9778 ± 0.0097	0.9767 ± 0.0104	0.9784 ± 0.0109	0.9798 ± 0.0100	0.9784 ± 0.0109
SVM	0.9045 ± 0.0082	0.9159 ± 0.0078	0.9041 ± 0.0084	0.9273 ± 0.0189	0.9329 ± 0.0163	0.9273 ± 0.0189	0.9335 ± 0.0167	0.9400 ± 0.0150	0.9335 ± 0.0167	0.9335 ± 0.0167	0.9400 ± 0.0150	0.9335 ± 0.0167
DT	0.9784 ± 0.0058	0.9810 ± 0.0055	0.9792 ± 0.0049	0.9864 ± 0.0092	0.9873 ± 0.0082	0.9864 ± 0.0092	0.9835 ± 0.0089	0.9845 ± 0.0082	0.9835 ± 0.0089	0.9830 ± 0.0086	0.9841 ± 0.0077	0.9830 ± 0.00086
NB	0.9938 ± 0.0028	0.9940 ± 0.0027	0.9937 ± 0.0028	0.9938 ± 0.0028	0.9941 ± 0.0028	0.9938 ± 0.0028	0.9938 ± 0.0028	0.9941 ± 0.0028	0.0038 ± 0.0028	0.9938 ± 0.0028	0.9941 ± 0.0028	0.9938 ± 0.0028
KNN	0.9733 ± 0.0106	0.9765 ± 0.0080	0.9731 ± 0.0105	0.9744 ± 0.0076	0.9775 ± 0.0063	0.9744 ± 0.0076	0.9744 ± 0.0076	0.9775 ± 0.0063	0.9744 ± 0.0076	0.9727 ± 0.0093	0.9760 ± 0.0076	0.9727 ± 0.0093
RF	0.9938 ± 0.0033	0.9942 ± 0.0029	0.9938 ± 0.0033	0.9949 ± 0.0033	0.9954 ± 0.0028	0.9949 ± 0.0033	0.9949 ± 0.0033	0.9954 ± 0.0028	0.9949 ± 0.0033	0.9927 ± 0.0056	0.9932 ± 0.0054	0.9927 ± 0.0056
Extra Tree	0.9909 ± 0.0061	0.9919 ± 0.0049	0.9909 ± 0.0061	0.9915 ± 0.0074	0.9924 ± 0.0064	0.9915 ± 0.0074	0.9920 ± 0.0049	0.9928 ± 0.0039	0.9920 ± 0.0049	0.9882 ± 0.0046	0.9890 ± 0.0044	0.9882 ± 0.0046
XGBoost	0.9847 ± 0.0085	0.9859 ± 0.0075	0.9846 ± 0.0085	0.9898 ± 0.0066	0.9906 ± 0.0059	0.9898 ± 0.0066	0.9892 ± 0.0079	0.9899 ± 0.0073	0.9892 ± 0.0079	0.9914 ± 0.0044	0.9919 ± 0.0041	0.9914 ± 0.0044
Stacking	0.9923 ± 0.0043	0.9927 ± 0.0033	0.9923 ± 0.0034	0.9905 ± 0.0027	0.9912 ± 0.0022	0.9905 ± 0.0027	0.9932 ± 0.0059	0.9935 ± 0.0056	0.9932 ± 0.0059	0.9948 ± 0.0023	0.9951 ± 0.0021	0.9948 ± 0.0023

**Table 5:** Comparison with existing methods.

Reference	Model	Dataset	Accuracy
Elbasi et al. (2023) [13]	NB Classifier	Kaggle Dataset with feature selection	99.46%
S.P. Raja et al. (2022) [9]	Bagging	Kaggle with MRFE feature selection	97.29%
Biplob et al. (2024) [3]	XGBoost	Kaggle Dataset	99.09%
Ramzan et al. (2023) [6]	KNN	Kaggle Dataset	97.81%
Kiruthika et al. (2023) [5]	IDCSO-WLSTM	Kaggle Dataset	92.68%
Bakthavatchalam et al. (2022) [12]	MLP	Kaggle Dataset	98%
Proposed	Stacking physics-informed NN model	Kaggle Dataset	99.50%

## Data Availability

The original data presented in the study are openly available in [kaggle] at [https://www.kaggle.com/datasets/atharvaingle/crop-recommendation-dataset] (accessed on 7 June 2025).
